# Vaginal Microbiota Diversity of Patients with Embryonic Miscarriage by Using 16S rDNA High-Throughput Sequencing

**DOI:** 10.1155/2020/1764959

**Published:** 2020-11-20

**Authors:** Linfen Xu, Lingna Huang, Chengying Lian, Huili Xue, Yanfang Lu, Xiujuan Chen, Yong Xia

**Affiliations:** ^1^Department of Gynaecology, Fujian Provincial Maternity and Children's Hospital, Fujian Medical University, Fuzhou, Fujian, China; ^2^Department of Key Laboratory for Prenatal Diagnosis and Birth Defect, Fujian Provincial Maternity and Children's Hospital, Fujian Medical University, Fuzhou, Fujian, China; ^3^Department of Laboratory, Fujian Provincial Maternity and Children's Hospital, Fujian Medical University, Fuzhou, Fujian, China; ^4^Department of Obstetrics and Gynecology, Fuzhou Maternity and Child Health care Hospital, Fuzhou, Fujian, China; ^5^Fujian Medical University, Fuzhou, Fujian, China

## Abstract

Embryonic miscarriage severely affects the life quality and physical and mental state of pregnant women. However, the detailed mechanism underlying embryonic miscarriage is not fully understood. This study is aimed at analyzing embryonic miscarriage. We collected samples from 25 normal pregnant women and 25 embryonic miscarriage patients of similar age to analyze microbiota isolated from the vagina. Crude examination of the vagina isolates showed that compared with the control group, 80% of the embryonic miscarriage group contained a significantly lower number of *Lactobacillus*, the major healthy microbe in the vagina. Furthermore, the levels of Th1 and Th2 secreted cytokine interleukin 2 (IL-2) and interleukin10 (IL-10), respectively, were examined. Results showed that the IL2 level was higher, and IL10 level was lower in the embryonic miscarriage group than in the control group, whereas the IL2/IL10 level was higher in the embryonic miscarriage group than in the control group. This finding suggested that the immune response was suppressed in the embryonic miscarriage group. To further dissect the microbiota of the vagina in the two groups, 16S rDNA sequencing was performed. Bioinformatics analysis showed that 1096 and 998 overlapped operational taxonomic units were identified from the embryonic miscarriage and control groups, respectively. At the genus level, the relative abundance of Fam_Finegoldia, Lac_Coprococcus_3, and Lac_Roseburia significantly differed in the embryonic miscarriage group. Overall, our analyses provided potential biomarkers for embryonic miscarriage and elucidated the causative relationship between microbiota and immune responses and may enable the possible diagnosis and therapeutics of early pregnancy loss.

## 1. Introduction

The Human Microbiome Project identified and characterized a healthy human microbiome in 2007 to understand the role of the microbiome in human health and disease [1]. As high-throughput sequencing techniques have been developed and have become affordable, numerous studies have been conducted analyzing the composition of the human microbiome and the relationship between microbiome and disease [2–5]. Microbiota is a complex community of microorganisms, including bacteria, fungi, virus, and eukaryotes, which play a crucial role in regulating host physiological processes and subsequent disease development [4, 6, 7]. Traditionally, the acquisition of microbiome in humans is considered to begin during and after birth via microbial exposure for the first time, as the fetus is kept in a sterile environment [8]. However, recent studies revealed that the fetus and its supporting system, including amniotic fluid and placenta, is not sterile, and the colonization of the gastrointestinal tract microbiome starts in the uterus [9–11]. The diversity and composition of the infant microbiome is mediated by internal and external stimulation, which constantly increases over time, whereas the completion of the gut microbiome colonization occurs approximately three years after birth [12–14]. The composition of the human microbiota is different across the body sites, and many factors, including age, genetics, environmental exposures, socioeconomic status, pregnancy status, and diet, have been identified to influence the variation in composition and function [15].

The human vagina is a passage for menstrual blood, sperm, and baby. The composition of vaginal microbiota is a key player in conception and pregnancy [16, 17]. Although the composition varies by ethnicity, age, and even among individuals [18], a healthy human vagina is dominated and protected from antimicrobial substances by *Lactobacillus* spp., which maintain normal vaginal pH (3.6 to 4.5) by producing lactic acid [19–22]. Vaginal microbiota diversity and richness are related to adverse pregnancy outcomes, such as preterm birth and miscarriage [23–25]. Miscarriage is a common pregnancy complication, which is defined as spontaneous pregnancy termination before 20 weeks of the pregnancy affecting 12%–24% of all pregnancies [26, 27]. Miscarriage can be subcategorized according to gestational age at the point of miscarriage: embryonic miscarriage and fetal miscarriage [28]. The first eight weeks of pregnancy called the embryonic period is crucial for the development and formation of the embryo. Embryologists have identified embryonic period as the organogenesis phase, and the development of the heart, which is the first organ, starts approximately 21 days from the last menstrual period (LMP) and ends at 70 days from the LMP [29]. When the organogenesis is finished at approximately nine weeks after conception, the fetal period starts and the embryo is referred to as fetus [30]. The miscarriage in early pregnancy before 12 weeks accounts for almost 80% of the miscarriage, which is caused by recurrent pregnancy loss, maternal age over 35, chromosomal abnormalities, and structural abnormalities [28]. However, in most cases, the reason of the spontaneous pregnancy loss in early pregnancy is still elusive [31]. Thus, in the present study, we are aimed at investigating the relationship between vaginal microbiota and embryonic loss.

High-throughput sequencing has been widely used to investigate microbial diversity and detect pathogenic microorganism in humans and animals [32, 33]. High-throughput sequencing has implemented a wide range of applications, such as RNA sequencing, whole-genome sequencing, and DNA-protein interaction assays, with cheaper price and less time consumption compared with the older Sanger-sequencing technologies [34]. The analysis of 16S ribosomal DNA (16S rDNA) sequencing has been used to understand bacterial diversity and composition from different health status and body parts in humans without cultivation [35–39]. 16S rDNA and rRNA analysis enabled the identification of microbiota in reproductive age women [40–42], women with preterm labor [43, 44], and menopause women [45–47]. However, microbial diversity of the vagina in embryonic miscarriage has not been investigated.

In this study, the vagina isolates were examined to evaluate populations using microscope and immune response by detecting IL2 and IL10 and two Th1 and Th2 marker cytokines. In addition, we have revealed the diversity of microbial communities in embryonic miscarriage women using 16s rDNA sequencing for the first time. These results will be useful to further understand the reason of embryonic miscarriage and decrease the percentage of pregnancy loss by early tests in therapy.

## 2. Materials and Methods

### 2.1. Study Participants and Specimen Collection

Participants were recruited from the Fujian Provincial Maternal and Children's Hospital. From October 2018 to March 2019, 25–30 patients with embryonic miscarriage were enrolled, and, in the same period, 25–30 voluntary normal early pregnancy cases were enrolled for the control group. The study protocol and informed consent form were approved by the Ethics Committee of the Fujian Provincial Maternal and Children's Hospital, and written informed consent forms were provided to all participants. The embryonic miscarriage pregnancy inclusion criteria were (1) stopped menstruation, smaller uterus size than gestational weeks, elevated urine or blood HCG, gestational sac confirmed by ultrasound, but gestational sac was smaller than gestational weeks, no embryo was seen in the sac or the heartbeat was not detectable; (2) age of the mother is 18–45, gestational age ≤ 12 weeks; (3) no intake of medication, which may affect fetal development, 3 months prior to pregnancy; (4) healthy mother with no adverse pregnancy history; and (5) signed informed consent. The exclusion criteria were (1) patient with endocrine diseases, infectious or transmitted diseases, and immune disorder; (2) patient with genetic problem and mental illness; and (3) not willing to participate. Vaginal swab has been isolated from each participant using sterile cotton-tipped swabs. The vaginal swab was inserted, and the lateral wall of the mid vagina was swabbed carefully by gynecologists. The swabs were immediately kept at a deep freezer for the subsequent analysis [48]. Vaginal lavage fluid was collected using a sterile saline solution. We transferred 5 mL of sterile saline into a disposable 5 mL sterile syringe, then rinsed the vaginal posterior ridge and repeated several times. The lavage solution was completely aspirated and transferred into a cryotube and placed in a −80°C refrigerator.

### 2.2. Vaginal Microecology Evaluation

Collected vaginal secretion was smeared, naturally dried, fixed, and stained with Gram's crystal violet solution. After staining, bacterial diversity and *Lactobacillus* density were observed under oil immersion field microscope. Bacterial density was divided into four levels according to the number of bacteria: grade 1 (+) as 1–3 kinds of bacterium; grade 2 (++) as 4–6 kinds of bacterium; grade 3 (+++) as 7–10 kinds of bacterium; and grade 4 (++++) as more than 10 kinds of bacterium. *Lactobacillus* concentration was categorized into four levels according to the number of *Lactobacilli* in each field of view, and 5–8 fields were observed. No *Lactobacillus* was recorded as (-); less than 1 was recorded as (+); 1–5 was recorded as (++); 6–30 was recorded as (+++); and more than 30 was recorded as (++++). Immune factors (IL-2, IL-10) level in vaginal lavage fluid were analyzed using Human IL-2 ELISA KIT (SEKH-0008, Solarbio, Beijing, China) and Human IL-10 ELISA KIT(SEKH-0018, Solarbio, Beijing, China) by microplate reader (DNM-9602, Beijing, China).

.

### 2.3. DNA Extraction and 16S rDNA Sequencing

Microbial DNA was isolated from vaginal swab using the QIAamp Fast DNA Stool Mini Kit (Qiagen, Valencia, CA, USA) following the manufacturer instructions and stored at the deep freezer before use. The V4 region of the 16S rRNA gene was amplified and sequenced using the PGM Ion Torrent (Thermo Fisher Scientific, Waltham, MA, USA). The primer sequences were as follows: F: GTGCCAGCMGCCGCGGTAA, R: GGACTACHVGGGTWTCTAAT. The PCR product was then collected and quantified using a QuantiFluor^TM^ fluorometer (Promega Corporation, USA). The purified amplification products were mixed in equal amounts and then sequenced to construct a sequencing library. The samples were sequenced using HiSeq3000/4000 (Illumina, Inc., USA).

### 2.4. Statistical Analysis

Metagenomic biomarker discovery and related statistical significance were assessed using relative taxonomic abundances analyzed according to the Linear Discriminant Analysis (LDA) effect size method52. In LEfSe, Kruskal–Wallis rank-sum test was used to identify features with significantly different taxa abundances among groups, and LDA to calculate the size effect of each feature. An alpha significance level of 0.05, either for the factorial Kruskal-Wallis tests among classes or for the pairwise Wilcoxon test between subclasses, and a size-effect threshold of 2.0 on the logarithmic LDA score were used for discriminative microbial biomarkers. For correlation analysis, microbiota abundance data were divided into independent data matrices (clearance group, persistence group, and HPV−group, as control). The correlation coefficients and significant negative correlations (*p* < 0.05) between *Lactobacillus* abundance data and all the other taxa were calculated using the pairwise Spearman's correlation and two-tailed probability of *t* for each correlation.

Data collation, screening, and statistical analysis were conducted using SPSS v20.0 (IBM) software: the metered data was recorded in *N* (%) or was presented as the mean ± standarddeviation (*x* ± *s*). The counting indicator was compared between groups using the card-side test, and the two sets of data were compared using the Mann-Whitney test, and *p* < 0.05 and *p* < 0.001 were considered statistically significant.

## 3. Results

The characteristics of the participants are shown in Table 1. No statistical difference was observed in age, days from LMP, number of pregnancies, and the size of the gestational sac between embryonic miscarriage and control groups (*p* > 0.05). Thus, the potential interferences of the individual factors could be eliminated.

### 3.1. Altered Concentration of *Lactobacillus* sp. and Diversity in Embryonic Miscarriage Pregnancy

Healthy vaginal microbiota is dominated by various *Lactobacillus* species, which are important in inhibiting the growth of microorganisms and preventing vaginal infectious diseases [49, 50]. Especially during the pregnancy, healthy microbiota is characterized by increased concentration in *Lactobacillus* species but decreased in diversity compared with nonpregnant women [51, 52]. Thus, the concentration of *Lactobacillus* and the diversity of vaginal microbiota were evaluated. The results showed that 56% of the embryonic miscarriage group population exhibited grade 2 *Lactobacillus* concentration, whereas 88% of the control group population showed grades 3 to 4 concentration levels (Table 2). In addition, the embryonic miscarriage group showed a significantly higher level of diversity compared with the control (Table 3). Overall, the embryonic miscarriage group showed significantly decreased *Lactobacillus* concentration and significantly increased diversity compared with normal control (both *p* < 0.001).

### 3.2. Th1 and Th2 Secreted Cytokine IL-2 and IL-10 Levels Showed Immunosuppression

In addition, vaginal local immunity was detected by measuring Th1 (T cell helper 1) and Th2 (T cell helper 2) cytokines. Under normal circumstances, Th1 and Th2 balance is a key factor of immune function. Th1 cells synthesize IL-2 and promote proinflammatory activation, whereas Th2 cells synthesize IL-10 and promote anti-inflammatory activation [53, 54]. Previous studies have reported abnormally upregulated IL-2, and downregulated IL-10 was observed in reproductive failure [55–57]. In agreement with a previous study, the results showed increased IL-2 (*p* < 0.05) and decreased IL-10 level in the embryonic miscarriage group compared with control (Table 4). IL-2/IL-10 ratio was also increased in the embryonic miscarriage group (*p* < 0.05). This resulted in suppressed immune system (Table 4).

### 3.3. Identification of Microbiota by Sequencing 16S rDNA

**Figure 1:**
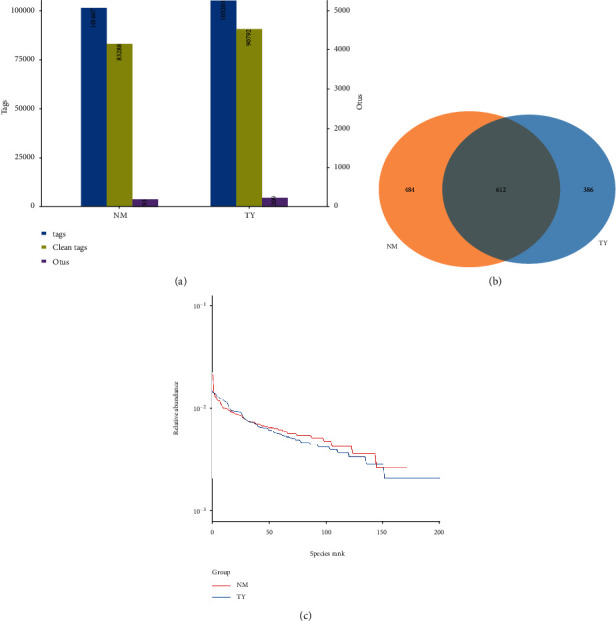
Analysis of 16S rDNA sequencing results. (a) Statistics of the tags and OTUs from the 16S rDNA sequencing results. Total tags (columns in blue) represent available sequence numbers; clean tags (columns in green) represent the valid sequence numbers; OTUs (column in purple) represent the final OUT numbers. (b) Overlapped operational taxonomic units (OTUs) between normal and embryonic death groups by Venn diagram. (c) Rank abundance curve of groups. *x*-axis represents the abundance species rank, and *y*-axis represents the relative abundance measured on the log scale.

Vaginal microbiota composition and diversity were compared by 16S rDNA sequencing. A previous study indicated the V4 regions have the greatest similarity with community profiles [58]. The V4 regions of the 16S rDNA gene were used for clustering operational taxonomic units (OTUs). Raw data generation was performed by the Illumina sequencing program. Impurities and tags with poor quality were removed, and effective tags were obtained. As shown in Figure 1(a), 101 607 sequencing tags were obtained from the control group, whereas 105 203 tags were obtained from the embryonic miscarriage group. We annotated 83 288 and 90 792 tags using QIIME (version 1.8.0) toolkit, respectively. All effective tags were clustered based on 97% sequence similarity threshold. On average, 93 and 209 OTUs were identified in the embryonic miscarriage group and control group, respectively.

The total amount of the OTUs was 1096 and 998 from the embryonic miscarriage group and control group, respectively. The common OTU numbers between the two groups were 612. Only 386 OTUs were unique in the embryonic miscarriage group, and 484 OTUs were unique in the control group (Figure 1(b)). The percentage of overlapped OTUs was 55.84% in the control group, whereas 61.32% in the embryonic miscarriage group. This result indicates that large portions of the vaginal microbiota were common between the embryonic miscarriage and control groups.

The rank abundance curve is shown in Figure 1(c). The rank abundance curve is used to visualize species richness and species evenness. Species richness can be represented by the number of different species, and the slope of the line in the graph can reflect the species evenness. Each line represents the OTU abundance distribution of the sample, and the length of the horizontal axis reflects the number of OTUs. In the present study, the case group abundance curve showed a steep gradient compared with the control group, which indicates lower in species similarity than the control group as the high-ranking species have much higher abundance than the low-ranking species.

### 3.4. The Composition of Bacteria and Relative Abundance or General Taxonomic Compositional Traits

**Figure 2:**
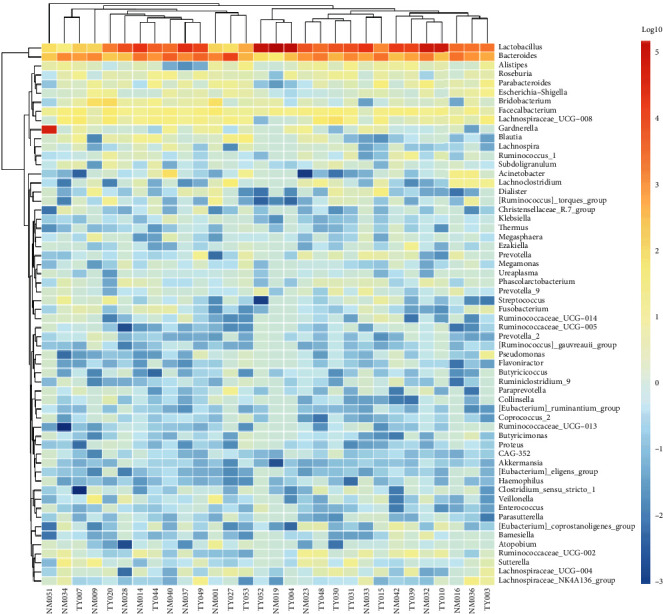
Heatmap clustering for vaginal microbiota at the genus level. Top 100 representative 16 s rRNA gene-based bacterial sequences classified at the genus level. Red indicates higher abundance, while green and blue represent progressively decreasing abundance.

**Figure 3:**
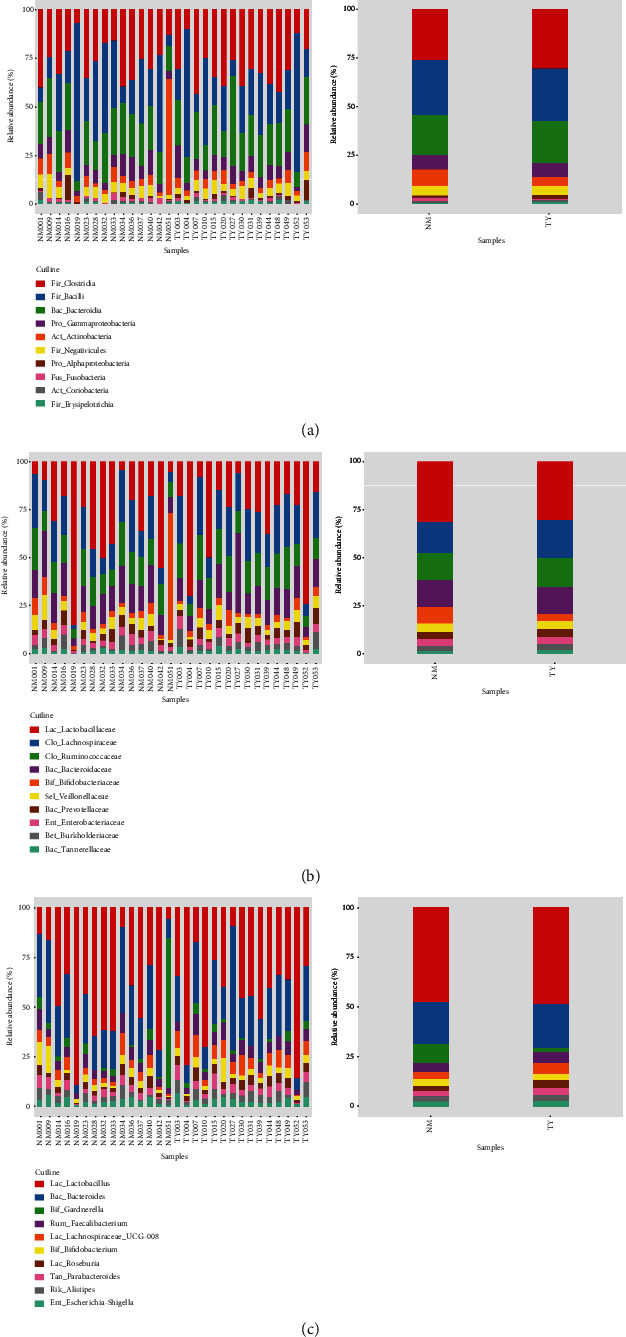
Relative abundance of vaginal microbiota show a significant difference between control and experimental group. (a) Class level; (b) Family level; (c) Genus level.

Through sequencing, the detection of bacteria and archaea in microbiota has become possible. Bacteria were predominant, whereas Archaea were detected only in three cases (two cases from control, one case from embryonic miscarriage). We detected a total of 278 phyla, 434 classes, 681 orders, 1167 families, and 2671 genera in the vaginal microbiota community from 15 embryonic miscarriage and control cases. To show the relative abundance of bacterial communities, we showed the heat map of the histogram (Figure 2). At class level, relative abundances of Fir_Erysipelotrichia (*p* = 0.011) and Fus_Fusobacteriia (*p* = 0.028) are significantly different between the two groups. At the family level, only Ery_Erysipelotrichaceae showed a significant difference (*p* = 0.012). At genus level, the significant differential bacteria were Fam_Finegoldia (*p* = 0.017), Lac_Coprococcus_3 (*p* = 0.010), and Lac_Roseburia (*p* = 0.007) (Figure 3).

### 3.5. Alpha Diversity and Beta Diversity

**Figure 4:**
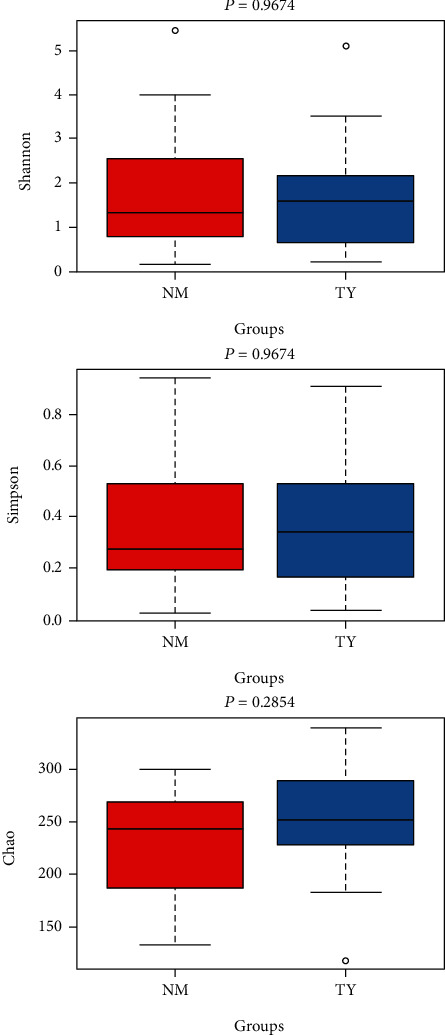
Alpha diversity index and rarefaction curve. Shannon, Simpson, Chao index in embryonic death group and control group. The box plots represent diversity measures and the central line represents the median value.

**Figure 5:**
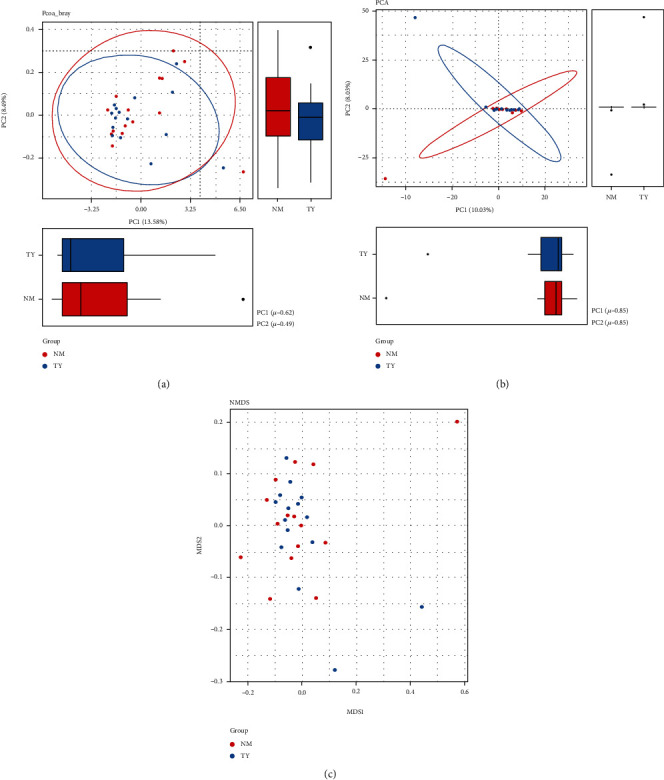
Beta-diversity comparison of microbiota in embryonic death group and control group. (a) PCoA, (b) PCA, and (c) NMDS.

Vaginal microbiota showed low alpha and beta diversity [59]. The alpha diversity represents the richness and diversity of the microbial community. Among diversity indexes, Chao exhibited that the observed species are more focused on the richness of the number of microbial communities, whereas Shannon and Simpson are more reflected diversity and evenness of the communities. Shannon, Simpson, and Chao indices showed no significant difference of the vaginal microbial community between the two groups (Figure 4, *p* > 0.05). Beta diversity represents the similarity of the microbial composition between samples. Nonmetric multidimensional scaling (NMDS), Principal Coordinate Analysis (PCoA), and Principal component analysis (PCA) results showed PC1 = 10.03% and PC2 = 8.03% (Figure 5, *p* > 0.05). Overall, alpha and beta diversity analysis between embryonic miscarriage samples and control showed no significant difference.

## 4. Discussion

Embryonic miscarriage is identified when the embryo is not seen in embryo sac or embryo is detected but with no cardiac activity using sonography [60], and the case of embryonic miscarriage has been increasing. The composition of the microbiota can be affected by several factors, such as lifestyle, food intake, medications, and immunity [61], which may result in pregnancy loss. However, the relationship between the microbiota and embryonic miscarriage is not clear.

To date, little research has been done on the effect of vaginal microbiota in pregnant women, particularly, those sporadic abortions before gestational age under 9 weeks. A similar study showed that the vaginal bacterial communities of 10 patients with unexplained recurrent miscarriage (RM) and 10 healthy volunteers were sampled and subjected to the bacterial 16S rRNA gene sequencing. At the genus level, Lactobacillus was the most dominant genus in the two groups. PCoA analysis suggested that changes in vaginal flora may be the cause of/associated with RM [25]. Another study indicates that first-trimester miscarriage associated with reduced prevalence of Lactobacillus spp.-dominated vaginal microbiota is classified using hierarchical clustering analysis, compared with viable pregnancies. Incomplete/complete miscarriage associated with higher proportions of Lactobacillus spp.-depleted communities is compared with missed miscarriage [62]. In our study, we are aimed at understanding the correlation between the diversity of vaginal microbiota and the embryonic survival and death. To analyze the mechanism of embryonic miscarriage, the 25 control individuals with pregnancy and 25 embryonic miscarriage patients with similar age, days from LMP, number of pregnancies, and the size of the gestational sac, were included to analyze vaginal microbiota to eliminate other factors for the collection of data. First, in the examination under the microscope, we identified that the population of *Lactobacillus*, which is the marker representing healthy microbiota, was significantly decreased in the embryonic miscarriage group than in the control group. Half of the embryonic miscarriage population showed grade 2 concentration, whereas most of the control group population showed grades 3 to 4 concentration, which suggests that the population of probiotics was decreased in the embryonic miscarriage group. Next, the immune response in the vagina of the two groups was evaluated. The interplay between microbiota and the host closely involves the immune system. In particular, the vaginal microbiota is classically characterized. A narrative review study systematically investigated the interplay between the immune system and microbiota in gynecological diseases; It suggested that a decreased concentration of Lactobacilli seems to be playing a role in preterm labor as well as the increased levels of proinflammatory cytokines, and immune response strictly interacts and strictly regulates microbiota itself [63]. Previous studies showed that abnormally upregulated IL-2 and downregulated IL-10 were observed in reproductive failure [55–57]. Our results showed that the level of IL2 was higher, whereas IL10 was lower in the embryonic miscarriage group than the control group, suggesting IL2 is dominant than IL10 that resulted in immune system depression in the embryonic miscarriage group. The results are consistent with others' studies. But, one study showed that no significant difference between the pregnant and nonpregnant cows was found in confidence regarding both alpha diversity and beta diversity [64].

To further dissect the total microbiota, the vagina isolate DNA was extracted, and 16S rDNA sequencing was performed. The sequencing data showed that a total of 101 607 and 105 203 sequencing tags were obtained from the control and embryonic miscarriage group, respectively. Further analysis using sequencing tags identified that 93 and 209 OTUs were isolated in the embryonic miscarriage control group, respectively. The total amount of the OTUs were 1096 and 998 from the embryonic miscarriage group and control group, respectively. Among them, 612 OTUs were common in the two groups, and 386 OTUs were specific to the embryonic miscarriage group, whereas 484 unique OTUs belonged to the control group, which suggests that the total vaginal microbiota were similar between the embryonic miscarriage and control groups. However, taxonomic analysis of the top 10 populations indicated that the population and type of microbiome were similar between two groups, with no significant differences. The top 10 microbiome were further classified by phylum, class, family, genus, and species for detailed evaluation of the microbiota. These results suggest that the relative abundance of microbiota was changed between the embryonic miscarriage group and control group, including Fir_Erysipelotrichia, Fus_Fusobacteriia, Ery_Erysipelotrichaceae, Fam_Finegoldia, Lac_Coprococcus_3, and Lac_Roseburia (Figure 3). Studies have compared the diversity of vaginal microorganisms in women with high-risk HPV infection and determined that the relative abundance of *Finegoldia* is one of six bacterial genera with statistical differences [65]. HPV infection has been reported as a risk factor of spontaneous abortion, and the risk level of different genotypes of HPV remains unchanged [66].

The results showed that the relative abundance of *Bacteroides* and gibberellinae could be used as biomarkers [67]. Some studies have found a certain correlation between spontaneous abortion and diabetes [68] to study the diversity of vagina microorganism during the period of genital tract infection and to find the vagina ecosystem in the period of common infection of the female genital tract. Vulvovaginal candidiasis (VVC), *Chlamydia trachomatis* (CT), and bacterial vaginosis (BV) are reproductive tract infection that are primarily characterized by anaerobes, such as *Gardnerella*, *Prevotella*, megastrobila, rosacea, and *Cyclospora* [69], which has been reported to increase the risk of miscarriage [70]. The change of microbiota may result in depression of the vagina environment and, subsequently, cause pregnancy loss. The population of patients indeed requires an increase to clarify the relationship between microbiota and embryonic miscarriage.

## 5. Conclusions

These results allow us to understand how vaginal microbiota protects women's health and embryo development or survival. Monitoring and control of the vaginal microbiota can be a potential protective approach of embryonic miscarriage in clinical practice.

## Figures and Tables

**Table 1:** Characteristics of study participants and control.

Characteristic	Control (NM, *N* = 25)	Embryonic miscarriage (TY, *N* = 25)
Age (years)	29.63 ± 4.85	28.53 ± 4.38
Days from LMP	53.52 ± 6.85	51.72 ± 5.23
Gravidity (times)	2.15 ± 1.10	2.36 ± 1.44
Gestational sac (cm)	1.62 ± 0.23	1.59 ± 0.19

The metered data were presented as the mean ± standarddeviation (*x* ± *s*), *p* > 0.05.

**Table 2:** Comparison of *Lactobacillus* concentration in vaginal microbiota.

	−	+	++	+++	++++
Control (*n* = 25)	0 (0%)	0 (0%)	3 (12%)	6 (24%)	16 (64%)
Embryonic miscarriage (*n* = 25)	5 (20%)	4 (16%)	14 (56%)	2 (8%)	0 (0%)

*Lactobacillus* concentration was categorized into four levels according to the number of *Lactobacilli* in each field of view and 5–8 fields were observed. No *Lactobacillus* was recorded as (-); less than 1 was recorded as (+); 1–5 was recorded as (++); 6–30 was recorded as (+++); and more than 30 was recorded as (++++). The metered data is recorded in *N* (%), *p* < 0.001.

**Table 3:** Comparison of diversity of the vaginal microbiota.

	+	++
Control (*n* = 25)	20 (85%)	5 (15%)
Embryonic miscarriage (*n* = 25)	8 (32%)	17 (68%)

Bacterial density was divided into four levels according to the number of bacteria: grade 1 (+) as 1–3 kinds of bacterium; grade 2 (++) as 4–6 kinds of bacterium; grade 3 (+++) as 7–10 kinds of bacterium; and grade 4 (++++) as more than 10 kinds of bacterium. The metered data is recorded in *N* (%), *p* < 0.001.

**Table 4:** IL-2 and IL-10 levels and the ratio of IL-2 : IL-10 (units: pg/ml).

	IL-2	IL-10	IL-2/IL-10
Control	87.43 ± 6.15	49.02 ± 3.35	1.79 ± 0.16
Embryonic miscarriage	98.440 ± 5.59	42.33 ± 2.43	2.33 ± 0.35

The metered data were presented as the mean ± standarddeviation (*x* ± *s*), *p* < 0.05.

## Data Availability

All data are in this article.
